# Can vouchers make a difference to the use of private primary care services by older people? Experience from the healthcare reform programme in Hong Kong

**DOI:** 10.1186/1472-6963-11-255

**Published:** 2011-10-07

**Authors:** Carrie HK Yam, Su Liu, Olivia HY Huang, EK Yeoh, Sian M Griffiths

**Affiliations:** 1 Division of Health System, Policy and Management, School of Public Health and Primary Care, The Chinese University of Hong Kong, Hong Kong

## Abstract

**Background:**

As part of its ongoing healthcare reform, the Hong Kong Government introduced a voucher scheme, intended for encouraging older patients to use primary healthcare services in the private sector, thereby, reducing burden on the overwhelmed public sector. The voucher program is also considered one of the strategies to further develop the public private partnership in healthcare, a policy direction of high political priority as indicated in the Chief Executive Policy Address in 2008-09. This study assessed whether the voucher scheme, as implemented so far, has reached its intended goals, and how it might be further improved in the context of public-private partnership.

**Methods:**

This was a cross-sectional study using structured questionnaires by face-to-face interviews with older people aged 70 or above in Hong Kong, the target group of the demand-side voucher program.

**Results:**

71.2% of 1,026 older people were aware of the new voucher scheme but only 35.0% had ever used it. The majority of the older people used the vouchers for acute curative services in the private sector (82.4%) and spent less on preventive services. Despite the provision of vouchers valued US$30 per year as an incentive to encourage the use of private primary care services, after 12-months of implementation, 66.2% of all respondents agreed with the statement that "the voucher scheme does not change their health seeking behaviours on seeing public or private healthcare professionals". The most common reasons for no change in their behaviours included "I am used to seeing doctors in the public system" and "The amount of the subsidy is too low". Those who usually used a mix of public and private doctors and those with better self-reported health condition compared to last year were more likely to perceive a change in their own health seeking behaviours.

**Conclusions:**

Our study showed that despite a reasonably high awareness of the voucher scheme, its usage was low. The voucher alone was not enough to realize the government's policy of greater use of the private primary care services. Greater publicity and more variety of media promotion would increase awareness but the effectiveness of vouchers in changing older people's behaviour needs to be revisited. Designating vouchers for use of preventive services with evidence-based practice could be considered. In addition to the demand-side subsidies, improving transparency and comparability of private services against the public sector might be necessary.

## Background

### Healthcare reform in Hong Kong

Hong Kong has a mixed healthcare system with both public and private sectors providing primary and secondary care services. Over 90% of all inpatient services (in terms of the number of bed days) are provided by public hospitals whereas around 70% of outpatient services are provided in the private sector [1]. The public services are largely funded by the Government through general taxation with small copayments at the point of care. The private health services are mainly from out-of-pocket household expenditure, and private insurance or employer-provided medical benefits play a relatively minor role [2]. Primary care in Hong Kong - fragmented and unsystematic - does not occupy a central position in the local healthcare system. Most patients indulge in "doctor-shopping" and holistic primary care especially preventive care based family-doctor model is not routine in Hong Kong [3]. Many studies in Hong Kong have also found that older people do not mind attending the publicly funded general outpatient clinics despite long waits and crowded conditions [4,5]. They are the main users of the public outpatient clinics services [6]. As a result of longevity, increasing occurrence of chronic diseases, as well as multiple morbidity and disability, the need and demand for healthcare services by the older population is growing and likely to expand. At the same time, the cost of healthcare is expected to increase, causing affordability concerns for both individuals and society [7-9]. Such double threats to the healthcare system are not unique to Hong Kong. The challenges of providing better primary care and healthcare financing are at the heart of many healthcare reforms, currently being carried out around the globe, the United States, the United Kingdom and Hong Kong included [1,10-12].

Indeed, the Chief Executive of Hong Kong, in his annual 2008-09 policy address, highlighted the importance of enhancing primary care in the ongoing healthcare reform, and introduced new policies to develop basic models for primary care services and promote public-private partnerships [13]. The Elderly Healthcare Voucher Scheme was one of these policies.

### Introduction of the voucher program and literature review

The idea of using vouchers as a financial incentive or lever to encourage behavioural change thus leading to bigger system change is not new. Specifically, vouchers are a type of demand-side consumer-led near-cash social transfer that can be redeemed for goods and services. Vouchers are commonly used in health and education services aiming at encouraging the use of under-consumed services, targeting beneficiaries, and increasing client satisfaction [14-16]. Different countries and continents have different reasons for introducing vouchers. As for the purpose of reducing some of the demand-side barriers to access (particularly costs), China Yunnan, Taiwan and Bangladesh had implemented maternal health voucher for the poor women to access quality maternal health services [14,17,18]. There were also schooling voucher for the poor girls in Pakistan and Bangladesh to encourage school enrolment [14]. In boosting demand for under-utilized services, Nicaragua had a sexually transmitted infections voucher scheme aiming at boosting the intake of treatment and prevention services for high risk groups such as commercial sex workers and their partners and clients [19]. France also had immunization voucher for asthmatic children in stimulating the low influenza vaccination coverage [20]. To promote abstinence from cigarette smoking, United States Baltimore had goods/services vouchers as incentive for ex-smokers to remain drugs-free [14].

As for the impact of different voucher programs, the evidence has been mixed. However, in general, the results of voucher schemes in specific health preventive services e.g. sexual and reproductive health services, child and maternal services, mammography screening, vaccination uptake, and medication compliance programs has been positive, especially in encouraging people to perform clearly defined, time-limited, simple behavioural tasks [20-27]. However, the objectives, deliverables and efficacy of a voucher system are contingent on how a supplementary financing option, such as voluntary private health insurance or medical savings account system, is structured [28]. Despite different reasons for setting up a voucher program, how successful it is depends on some common factors, including the target groups, mechanism of vouchers, and the current functioning of health and education sector [14,15].

**Figure 1:**
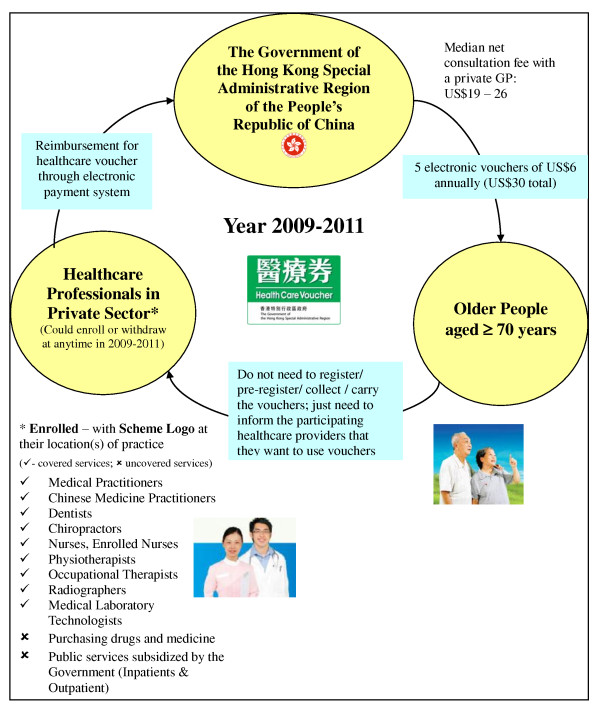
**Details of the elderly healthcare voucher scheme**.

The Hong Kong Government launched the 3-year elderly healthcare voucher scheme in January 2009, aiming to provide choices (including both curative and preventive care) for older people in addition to the existing public primary care service, thus reducing their reliance on public healthcare services (public-private imbalance), providing better access to care and promoting the concept continuity of care for the older people. More specifically, the program provides older people aged 70 or above (with around US$1,426 household income on average) five US$6 healthcare vouchers annually (US$30 total) to partially subsidize their use of private primary care services. Private doctors in Hong Kong charge different prices (around US$19-26 per consultation compared to a consultation fee of US$6 in a public outpatient clinic) for mainly curative care on an episodic basis. Beneficiaries do not need to pre-register or to collect healthcare vouchers themselves. Healthcare professionals in the private sector volunteer to enrol in the scheme. There is a computerized electronic system in the Government in operating the voucher scheme. Once the healthcare professionals enrolled, they could use this electronic system to register and create the personal voucher accounts for each eligible patient, and for their reimbursement of vouchers. Details of voucher scheme are shown in Figure 1.

### Motivation and Study Objectives

Our study was, of necessity given the political context and sudden policy announcement, carried out as the voucher scheme was introduced. The voucher scheme was deemed to fit well into two priority areas on the Hong Kong Government's agenda - primary care and public-private partnership and was introduced in the Chief Executive's policy address. We are not aware of studies done *a priori *to estimate the appropriate subsidy amount, or simulate the program's potential impact before its launch. The lack of evidence was indeed the main motivation behind our current 'real-time' study. Our interest, as well as that of government who funded us, was whether this new policy - the introduction of a voucher scheme (as implemented) - had affected the older population's health seeking behaviours and shifted them toward the Government's desired healthcare reform direction of greater use of primary healthcare services in the private sector. More specifically, our main objective of the study was to assess whether the voucher program so far has realized its intended goals, and to provide lessons learned (i.e. the missing evidence before launching the program) for future program improvement and policy changes.

## Methods

### Study design and subjects

A cross-sectional survey was conducted one year after the launch of the voucher scheme (January-June 2010) among older people aged 70 or above who are eligible for the elderly healthcare voucher scheme in Hong Kong. Since there is no population register from which we could randomly sample older people in Hong Kong, we used a convenience sampling to recruit two groups of older people: (i) older people who were sick and were attending outpatient clinics in either public or private sectors, and (ii) older people who were generally well at the time of enumeration surveyed either in the parks while doing morning exercise or in the elderly health centres during their physical check up. Exercising in the parks is part of normal culture for older Chinese people living in urban environments. The elderly health centres are run by the Government with an aim to enhancing primary healthcare by providing health assessment, physical check up, counselling, curative treatment and health education to older people aged 65 or above with an enrolment fee of US$14 per year. The 3 selected public parks were chosen in the districts with higher, medium and lower average household income. The less healthy older people were recruited from 2 public general outpatient clinics and 12 private clinics in various districts in Hong Kong. Data were collected face-to-face by trained interviewers using a structured questionnaire. For some of the private clinics cases were interviewed over the phone using contact information provided by the doctors with consents obtained from the patients in advance.

### Questionnaire

The questionnaire consisted of four sections: (1) Demographic characteristics, and healthcare services utilization pattern, (2) Awareness of the scheme e.g. whether the older people were aware of the scheme and channels to know about the scheme, (3) attitudes: (a) value assessment - whether the older people agreed that "the voucher is useful", "convenient to use", "the subsidy amount is enough", and "the coverage of services under the scheme is sufficient" and (b) perceived change of behaviours - whether the voucher scheme would encourage the use of private primary care services more than before and whether voucher scheme would change health seeking behaviours on where to see healthcare professionals, (4) Voucher usage, e.g. whether they have ever used the vouchers for private primary care services, reasons for not using it, types of professionals and medical services ever used for the voucher. English version of questionnaire used is available as additional file 1.

### Outcome measures

The primary outcome of the study was the changes in perceived health seeking behaviour - measured by asking the older people if *they thought there had been a change *in their health seeking behaviour when they sought advice from healthcare professionals after the introduction of the voucher scheme. We also assessed who were the users of voucher scheme - measured by asking whether the older people *had ever used *vouchers to see private primary care professionals (which signals actual behaviour change especially for those who are used to seeing public doctors).

### Statistical analysis

Descriptive statistics were collected on the awareness, attitudes and usage of voucher scheme. Univariate analysis of (i) perceived changes in health seeking behaviour and (ii) use of vouchers was undertaken. The variables that were significant in the univariate analysis were tested by logistic regressions to identify predictors of perceived behaviour change and factors associated with the use of vouchers and to estimate adjusted odds ratio (OR) with 95% confidence intervals (CI).

### Ethics

Ethical approval was obtained for the study from the ethics committees in the Hong Kong Hospital Authority and Department of Health.

## Results

### Respondents' profile and health status

In total, 1,026 older people were successfully interviewed with a response rate of 79.2%. 57.6% were generally well at the time of interview: 28.0% recruited from the public parks and 29.6% from the elderly health centres. 42.4% were less healthy at the time of interview and were recruited from public general outpatient clinics (31.2%) and private clinics (11.2%). The mean age was 78 years (standard deviation of 6 years) and 42.6% were male, which was similar to those of the general population [29]. More than a third (35.7%) had received no education. 42.7% had a monthly income below US$1,282 (the median monthly household income for a domestic household with older people is US$1,426 in 2006) [30]. 9.9% were receiving comprehensive social security assistance (a financial allowance of US$313 to US$567 per month given by the Government to those unemployed or low income family). This sampling possibly under-represented wealthier older people but since the aim of the voucher scheme is to encourage people who rely on the public sector (more likely to be poor) to use more private services, this does not detract from our results.

Most of the older people usually consulted both public and private doctors for their healthcare prior to the launch of the voucher scheme (44.3%). 34.1% were used to seeing public doctors in the outpatient clinics only while 19.6% sought care from private doctors only. The majority did not have private health insurance or medical benefits (93.5%). The most common self-reported chronic diseases were high blood pressure (61.6%) and diabetes (23.7%). 41.5% felt their general health condition was worse than last year and 46.9% said it was about the same. Demographics of all respondents are shown in table 1.

**Table 1:** Characteristics of respondents, and univariate association of variables affecting (i) perceived change of health seeking behaviours and (ii) use of vouchers upon the introduction of voucher scheme

		(i) Perceived change Whether changed health seeking behaviours			(ii) Use of voucher Whether ever used the vouchers		
**Variable**	**Total**	**Yes**	**No**	**P value of difference**	**Yes**	**No**	**P value of difference**

**Demographics**							
**Sex**							
Men	437 (42.6)	118 (29.9)	277 (70.1)	0.058	147 (35.1)	272 (64.9)	0.408
Women	589 (57.4)	129 (24.3)	402 (75.7)		212 (37.7)	351 (62.3)	
**Age**							
70 - 79	636 (62.2)	158 (26.9)	430 (73.1)	0.599	223 (36.3)	392 (63.7)	0.577
80 - 89	345 (33.7)	79 (25.8)	227 (74.2)		125 (37.3)	210 (62.7)	
90 or above	42 (4.1)	10 (34.5)	19 (65.5)		8 (27.6)	21 (72.4)	
**Marital status**							
Single	42 (4.1)	10 (27.8)	26 (72.2)	0.035	13 (31.7)	28 (68.3)	0.724
Married	661 (64.7)	178 (29.3)	429 (70.7)		233 (36.5)	406 (63.5)	
Divorced/Separated Widowed	319 (31.2)	59 (21.1)	221 (78.9)		113 (37.9)	185 (62.1)	
**Education level**							
No education	366 (35.7)	70 (21.5)	256 (78.5)	0.003	156 (44.4)	195 (55.6)	0.002
Primary	378 (36.9)	109 (31.8)	234 (68.2)		121 (33.3)	242 (66.7)	
Secondary	211 (20.6)	58 (30.1)	135 (69.9)		62 (31.5)	135 (68.5)	
Tertiary or above	70 (6.8)	10 (15.9)	53 (84.1)		20 (28.6)	50 (71.4)	
**Monthly household income**							
No income	85 (8.3)	17 (22.1)	60 (77.9)	< 0.001	21 (26.3)	59 (73.8)	< 0.001
HK"tabcaption"-9,999	352 (34.4)	61 (18.7)	265 (81.3)		163 (46.3)	189 (53.7)	
HK$10,000 or above	101 (9.9)	25 (26.6)	69 (73.4)		34 (34.0)	66 (66.0)	
Don't know/Not willing to answer	486 (47.5)	144 (33.7)	283 (66.3)		139 (31.0)	309 (69.0)	
**Social security assistance**							
Had social security assistance	102 (9.9)	27 (30.3)	62 (69.7)	0.411	35 (35.7)	63 (64.3)	0.855
**Living status**							
Lived alone	222 (21.7)	51 (26.0)	145 (74.0)	0.792	77 (37.7)	127 (62.3)	0.654
**Private health insurance**							
Had private health insurance	66 (6.5)	10 (17.2)	48 (82.8)	0.088	25 (41.0)	36 (59.0)	0.442
**Health condition**							
Self-rated health compared with last year							
Better	118 (11.5)	52 (49.1)	54 (50.9)	< 0.001	28 (25.7)	81 (74.3)	< 0.001
Similar	481 (46.9)	109 (25.5)	318 (74.5)		147 (32.2)	309 (67.8)	
Worse	426 (41.5)	85 (21.7)	307 (78.3)		184 (44.1)	233 (55.9)	
**Chronic condition**							
Had Diabetes	243 (23.7)	48 (22.3)	167 (77.7)	0.100	78 (34.2)	150 (65.8)	0.401
Had high blood pressure	632 (61.6)	143 (24.9)	431 (75.1)	0.122	231 (37.7)	382 (62.3)	0.345
**Medical consultation**							
Usually go to see which types of doctors before launch of voucher scheme							
Public doctor only	349 (34.1)	69 (21.0)	260 (79.0)	< 0.001	82 (23.6)	265 (76.4)	< 0.001
Private doctor only	201 (19.6)	41 (22.2)	144 (77.8)		98 (49.0)	102 (51.0)	
Both public and private doctor	454 (44.3)	133 (33.3)	267 (66.8)		176 (41.6)	247 (58.4)	
Don't know	20 (2.0)	4 (36.4)	7 (63.6)		8 (80.0)	2 (20.0)	
**Medical consultation in the past one month **(excluding the current episode for older people recruited in clinics)							
Had medical consultation	421 (41.2)	88 (22.6)	302 (77.4)	0.018	199 (47.6)	219 (52.4)	< 0.001
**Had hospitalization in the past one year**							
Had hospitalization	200 (19.8)	48 (26.2)	135 (73.8)	0.946	89 (45.4)	107 (54.6)	0.004

Values are numbers (percentages) of respondents.

Summation of respondent in saying "yes" and "no" on the questions of perceive change/use of voucher is not equal to the total number of respondent since we exclude those saying "don't know"/"don't remember"; P-value indicates significant difference between the perceived change/use of voucher and each independent variable.

### Awareness

71.2% of all respondents were aware of the elderly healthcare voucher scheme. Older people who were sick at the time of interview (74.5%) had a significantly higher awareness than those of generally-well older people (68.9%). The most common way of knowing about the scheme was from television advertisements (57.8%). However, less than half (46.8%) felt the amount of information was fair or sufficient.

### Attitudes towards the design features of the voucher scheme

**Figure 2:**
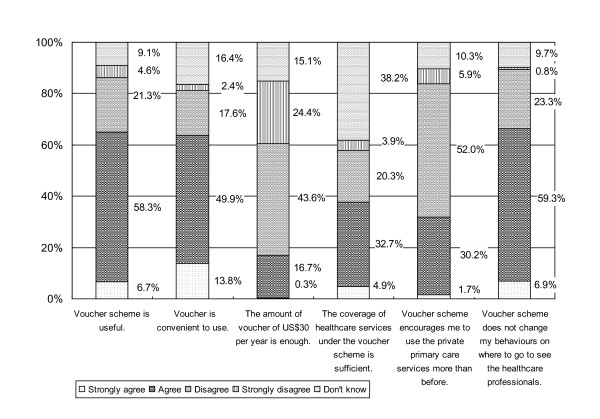
**Attitudes of the older people towards the voucher scheme**.

More than 60% agreed that the "voucher is useful" and "convenient to use". However, only 17% agreed "the amount of US$30 per year is enough". 35.7% suggested increasing the subsidy amount to US$38-64 and 31.7% to US$65-128. About 40% do not know whether "the coverage of services under the scheme is sufficient", perhaps because pricing in the private sector is unpredictable. The descriptive statistics on attitudes of respondents towards vouchers are summarized in Figure 2.

### Perceived change of health seeking behavior

Regarding the impact of voucher scheme on older people's health seeking behaviours, 66.2% said that "the scheme does not change my behaviours on where to see the healthcare professionals". There was similar resistance to change in response to the question on whether the voucher scheme encourages use of private primary care services more than before (only 31.9% agreed to the statement). The common reasons for not changing their behaviours included "I was used to seeing doctors in the public system" (26.0%) and "The amount of the subsidy is too low" (24.1%).

### Usage

Among all respondents, 35.0% had actually used voucher scheme. This percentage is close to the official statistics from the Government on the voucher claims (32% as at February 2010) [31]. The percentage of older people who had made use of their vouchers varied by the type of doctors they usually visited. Only 23.6% of the older people who usually visited public doctors had made use of their vouchers. For those who usually visited private doctors, almost half (49.0%) had made use of their vouchers during consultation. Reasons for not using vouchers were "limited choices of healthcare professionals because the healthcare professionals whom I usually see have not enrolled in the scheme" or "there are no enrolled healthcare professionals nearby" (46.4%), "I am used to seeing public doctors" (23.5%), and "I do not need to consult any healthcare professionals" (23.2%). Most of the older people used the vouchers for Western medicine doctors (87.5%), followed by Chinese medicine practitioners (11.4%) and dentists (5.0%). The vouchers were mainly used for acute curative services (82.4%) and not for preventive services such as regular follow-up for chronic conditions (7.0%) and dental services (4.5%), as the Government originally intended.

### Predictors on perceived behaviour change and use of vouchers

Univariate associations of (i) perceived health seeking behaviour change and (ii) use of vouchers with the personal characteristics are shown in table 1. In the logistic regression model of perceived behaviour change, those who were used to seeing both public and private doctors were more likely to perceive a change after the introduction of the voucher scheme, compared with those seeing public doctors only (OR: 1.65; CI: 1.15 to 2.38). Those with better self-reported health conditions compared to last year (OR: 2.11; CI: 1.28 to 3. 48) were also more likely to perceive a change in health seeking behaviours (table 2). With regard to the use of vouchers, those with no formal education relative to tertiary level or above (OR: 1.86; CI: 0.97 to 3.56) and those who were used to seeing both private and public doctors (OR: 2.58; CI: 1.84 to 3.61) or seeing private doctors only before the launch of voucher scheme (OR: 3.11; CI: 2.09 to 4.64) relative to those used to seeing public doctors were more likely to use vouchers. On the other hand, those self reporting better health compared to last year (OR: 0.54; CI: 0.32 to 0.92), without medical consultation in the past one month (OR: 0.45 CI: 0.34 to 0.60) or without hospitalization in the past one year (OR: 0.69 CI: 0.48 to 0.99) were less likely to use the vouchers (table 2).

**Table 2:** Multiple logistics regression model for (i) perceived change of health seeking behaviours, and (ii) use of voucher

	(i) Perceived change Whether change of health seeking behaviours	(ii) Use of voucher Whether ever used the vouchers		
**Variable**	**Adjusted odds ratio** **(95% CI)**	**P value**	**Adjusted odds ratio** **(95%CI)**	**P value**

**Marital status**				
Single	1.22 (0.54 - 2.78)	0.397	-	
Married	1.30 (0.89 - 1.90)		-	
Divorced/Separated Widowed	1		-	
**Education level**				
No education	1.70 (0.77 - 3.75)	0.051	1.86 (0.97 - 3.56)	0.020
Primary	2.46 (1.15 - 5.25)		1.13 (0.56 - 2.13)	
Secondary	2.21 (1.02 - 4.81)		1.13 (0.59 - 2.20)	
Tertiary or above	1		1	
**Self-rated health compared with last year**				
Better	2.11 (1.28 - 3.48)	0.003	0.54 (0.32 - 0.92)	0.046
Similar	0.94 (0.64 - 1.34)		0.77 (0.56 - 1.05)	
Worse	1		1	
**Usually go to see which types of doctors before launch of voucher scheme**				
Public doctor only	1	0.024	1	< 0.001
Private doctor only	1.03 (0.65 - 1.65)		3.11 (2.09 - 4.64)	
Both public and private doctor	1.65 (1.15 - 2.34)		2.58 (1.84 - 3.61)	
Don't know	2.09 (0.54 - 8.01)		1.38 (0.26 - 6.94)	
**Medical consultation in the past one month **(excluding the current episode for elders recruited in clinics)				
No	1.28 (0.91 - 1.76)	0.156	0.45 (0.34 - 0.60)	< 0.001
Yes	1		1	
**Hospitalization in the past one year**				
No	-		0.69 (0.48 - 0.99)	0.041
Yes	-		1	

*Adjusted for age, gender and living districts. Only those factors that were significant in the univariate analysis were tested by logistic regression*.

## Discussion

Our study explored the impact of introducing a voucher scheme as part of healthcare reform to encourage greater use of private primary care services. Despite a reasonably high awareness of the voucher scheme, its usage was low. The impact of the voucher scheme on its primary target group, the frequent users of public outpatient services, was relatively small (only 21.0% of those usually see public doctors felt there was a change in their health seeking behaviours). The voucher alone was not enough to stimulate the Governments objective of greater use of the private primary care services. Those who were healthiest tended to be the most likely to consider changing their behaviour and those who were already using the private sector were the most likely to use the vouchers.

International studies have shown that voucher schemes are generally effective when used for specific targeted health services especially in the uptake of preventive measures. For example, studies in Nicaragua and France demonstrated that vouchers boosted the uptake of sexual & reproductive healthcare services and vaccination respectively [20-22]. Evidence for the effectiveness of financial incentives was the strongest in drug misuse programmes [32]. A World Bank study pointed out that voucher schemes are often aimed at under-utilized services and are most effective if targeted at specific groups [16]. Another study by the King's Fund found that vouchers are effective in encouraging participation in simple behavioural tasks as well as lifestyle programmes [27]. In our study, the elderly healthcare voucher scheme covers all the primary healthcare services including curative and preventive services in the private sector. Its focus is on the use of subsidized private primary care services in general, but not targeted at the under-utilized preventive services, which might partly explain why the voucher scheme in Hong Kong failed to induce any noticeable behavioural change amongst the users of primary health care services during the first year of the pilot period. There is little evidence worldwide on whether a voucher scheme could incentivize the use of primary care services and development of family doctor model of care in the private sector. Our study therefore provides the insight that a general voucher scheme as currently designed was not effective in incentivizing the use of private primary care services among the older people in Hong Kong, who are used to receiving much more affordable services from the public sector.

Our findings also showed that not only did the older people in our study not perceive a change of their health seeking behaviours upon the introduction of voucher scheme, but there was a low level of actual voucher usage in the private sector for primary care services (only 35.0% of older people had made use of vouchers). Older people who are used to seeking care from private doctors are more ready and prepared than those relying on the public healthcare system to make use of healthcare vouchers. Those older people who were used to seeing public doctors were less likely to use the vouchers (23.6%) compared to those used to seeing private doctors only (49.0%) or a mix of public and private doctors (41.6%). The main reasons given were that they did not wish to change their usual practice of seeing public doctors and that the subsidy amount is relatively low. This not only indicated that the older people are content with services currently received in the public sector, despite long waits and crowded conditions, but in a large part this might reflect their low willingness to pay, perceived inability to pay and uncertainty about the price and quality of services provided in the private sector. A separate study was being conducted among the older groups on their willingness to pay for private sectors. In addition to the demand-side subsidies, making the services and prices in the private sector more transparent and comparable with public sector might help patients in making better informed decisions. This study has provided important early insight into the impact of the voucher scheme among the target group. Presentation to policymakers has suggested that they might wish to consider introducing more cost-effective incentives by targeting other subpopulations or specific services.

Furthermore, since the current usage of vouchers is low and the older people mainly use them for acute conditions, attempts to encourage use of private services for maintenance or control of their chronic diseases needs review, as does potential use of vouchers for promoting other evidence-based programmes such as care supported by guidelines. The small proportion (7.0%) of health care vouchers used on preventive services indicated that most older people give preventive services a low priority when it comes to healthcare spending decisions. In Hong Kong, only 2.5% of the entire health expenditure is spent on disease prevention and health promotion [33]. Further consideration should be put into designing vouchers for designated use for preventive services with evidence-based practice (such as cancer screening, hypertension or diabetes management) as this would address the unmet need that is known to exist, particularly since evidence from countries around the world has shown that primary care oriented health systems produce better health outcomes [34]. Also, it requires the concerted efforts of the government, healthcare service professionals and the media to gradually induce a cultural change that puts more value and emphasis on preventive care. In addition, our study showed that older people usually see both public and private doctors as well as attend both Western trained and Traditional Chinese medicine practitioners when they are sick, implying a doctor shopping behaviour without a continuous doctor-patient relationship. One of the aims of the voucher is to promote the model of continuity of care from a family doctor. Our study does not provide information on whether the patients will build up this continuous doctor-patient relationship with the use of vouchers. However, government statistics showed that there are early results implying that older people tend to stay with the same private doctors if they use vouchers. Further study is needed to examine the effects of voucher on this aspect of the reforms.

As part of healthcare reform to promote greater use of subsidized private primary healthcare services, the voucher scheme still has room for improvement to make it more effective. There appears to be a lack of interest in the voucher scheme from both supply and demand side. Greater publicity and more variety of media promotion and approaches would increase awareness and usage. Also, given only half of the registered private Western medicine doctors have enrolled in the voucher scheme [30], more healthcare professionals should be encouraged to enrol in the scheme to provide more choices for the older people. In addition, the level of subsidy should be reviewed since nearly 68.0% said the subsidy was not enough. Proper management and monitoring of voucher schemes is also necessary to ensure the actual consultation charges would not be increased by the voucher scheme. In our study, nearly half (44.8%) of the older people did not feel that there had been an increase in consultation fees subsequent to the launch of the voucher scheme, while 13.7% perceived an increase, and the rest (41.7%) said that they did not know. Further study is needed among the supply side to ascertain the range of co-payment charged by healthcare service professionals and whether the fees are beyond the willingness-to-pay of the older people. Reasons for the low participation rate of healthcare professionals should also be examined. Another aspect of the voucher scheme is its high transaction and administrative cost. Over-servicing might also occur because of the direct link between outputs and subsidies. The above factors might affect the effectiveness of the voucher scheme. Thus, any improvements should consider a feasibility assessment covering client expectation, support or enrolment from services providers, administrative and transaction costs, and accurate determination of price to ensure the efficiency of the voucher scheme [14].

Apart from the demand-side subsidy, other incentives such as supply-side subsidy might also be considered in encouraging primary care and improve the quality of care e.g. pay-for-performance using the Quality and Outcomes Framework in United Kingdom. The private sector plays a critical role in healthcare services provision. A proper public-private partnership model should be examined to make better use of resources in both the public and private sectors and to provide greater choice of services for individuals in the community.

One technical limitation we faced was getting a representative sample of the target population. We chose a convenience sample by recruiting both healthy and sick older people from parks and clinics because there is no good way of getting a population-based study sample in Hong Kong. Primary care doctors do not have unique records for their patients and it is common for patients to adopt doctor shopping behaviours. However we recruited older people from different districts in Hong Kong to reflect different socio-economic characteristics and we did confirm that the age and sex distribution of respondents are similar to those of the population. However, a household survey with participants randomly selected from the list of household addresses would ideally provide a more representative sample. Also, it was difficult to recruit from private clinics and 80% of the participating private clinicians in our study had joined the voucher scheme, which might lead to an over-estimate of voucher usage among this subgroup of respondents.

## Conclusions

Our study provides information about the impact of a policy change, the voucher scheme, and fills a knowledge gap about whether the policy change promoted its desired objective of greater use of the private sector in primary care. It also provided evidence for suggestions for improvement of the voucher scheme. Since many countries, including United Kingdom and United States, start to consider the use of financial incentives to promote changes in patients' behaviour, evidence about the effectiveness of vouchers is important. Hong Kong's recent experience provides an opportunity for others to draw lessons for healthcare reform in their own countries.

## Competing interests

The authors declare that they have no competing interests.

## Authors' contributions

All authors carried out and designed the study. CHKY wrote the first draft of the manuscript and all authors made important contributions to the subsequent draft. All authors have seen and approved the final version.

## Pre-publication history

The pre-publication history for this paper can be accessed here:

http://www.biomedcentral.com/1472-6963/11/255/prepub

## Supplementary Material

Additional file 1 **English version of questionnaire**. A copy of the English version of questionnaire used at the survey. Click here for file
