# Supplementary material for: Can vouchers make a difference to the use of private primary care services by older people? Experience from the healthcare reform programme in Hong Kong
Source: BMC Health Serv Res. 2011 Oct 7;11:255. doi: 10.1186/1472-6963-11-255 (PMC3200178; doi:10.1186/1472-6963-11-255)
Supplement: Additional file 1 — English version of questionnaire. A copy of the English version of questionnaire used at the survey. [file 1472-6963-11-255-S1.PDF]

Questionnaire A (Aged 70 or above) EHC/ GP/ Park/ GOPC: ________

Hi! I'm an interviewer from the School of Public Health and Primary Care, The Chinese University of Hong Kong. We are now conducting a study on the elderly healthcare voucher scheme launched by the Department of Health. I cordially invite you to participate in the interview. All information provided will be anonymous, kept confidential and will be used for research purposes only.

	**Screening Questions**	
1.	Are you aged 70 or above?	1- Yes: (Probe: years old)<br>2- No - End the interview
	**Awareness and Knowledge**	
	● ***Awareness of voucher scheme***	
2.	Are you aware of the elderly healthcare voucher scheme?	1- Yes<br>2- No (Skip to Preamble before Q11)
3.	How do you know about the voucher scheme? Any more?<br>*(Please check all that apply)*	1- TV advertisements<br>2- Press/ Magazines<br>3- Posters/ leaflets<br>4- Internet browsing<br>5- Words of mouth from family/ relatives/ friends<br>6- Elderly centres/ health talks<br>7- Radio advertisements<br>8- Healthcare professionals who have joined the voucher scheme<br>9- Others, please specify: ____________________
4.	**4a.** Do you know which healthcare professionals could join the voucher scheme?	1- Yes<br>2- No (Skip to Q5)
	(For those who knew which healthcare professionals could join)<br>**4b.** Please tell me the healthcare professionals whom you think have joined the scheme. Any more? **(Do not read out)**<br>*(Please check all that apply)*	1- Registered medical practitioners<br>2- Registered Chinese medicine practitioners<br>3- Registered dentists<br>4- Registered chiropractors<br>5- Registered nurses, enrolled nurses<br>6- Registered physiotherapists<br>7- Registered occupational therapists<br>8- Registered radiographers<br>9- Registered medical laboratory technologists<br>10- Others, please specify:______________________
	**4c.** How do you know which healthcare professionals has joined the voucher scheme? Any more?<br>*(Please check all that apply)*	1- Looking at the Scheme logo at their location of practice<br>2- Going to internet to check the lists of enrolled healthcare professionals<br>3- Words of mouth from family/ relatives/ friends<br>4- Others, please specify: ____________________

5.	**5a.** Do you feel the information of the voucher scheme provided to you is sufficient or not?	1- Very sufficient<br>2- Quite sufficient<br>3- Fair<br>(1–3: Skip to Q6)<br>4- Not quite sufficient<br>5- Very Insufficient<br>6- Don't know
	<u>*(For those who felt information not sufficient)*</u><br>**5b.** What kind of information you would like to enrich? Any more?<br>*(Please check all that apply)*	1- Channels to disseminate the lists of enrolled healthcare professionals<br>2- How to use the voucher<br>3- How to check my voucher account balance<br>4- Others, please specify: ____________________
	● ***Understanding on voucher scheme***	
	We would like to explore how much you know about the voucher scheme. Please tell us whether you think the following statements are correct or not.	

		Yes	No	Don't know
6.	Are you given 5 vouchers valued $50 each every year? *(Yes)*	1	2	3
7.	Could the vouchers be used for the Accident & Emergency services and the General/ Specialists out-patients services provided by the Hospital Authority? *(**No**: Voucher can be used only for private primary care services)*	1	2	3
8.	Could you use the vouchers for inpatient day surgery, e.g. cataract surgery? *(**No**: Voucher cannot be used for inpatient services)*	1	2	3
9.	Could the vouchers be used to purchase drugs/ medicine at community pharmacists? *(No)*	1	2	3
10.	Do you need to pre-register, pre-collect and carry the healthcare vouchers? *(No)*	1	2	3
	**Attitudes**			

<u>Preamble for those who are not aware of voucher scheme</u>:

"If you are aged 70 or above during the period from 2009 to 2011, holding a valid HK Identity Card or Certificate of Exemption, you will be provided with 5 vouchers of $50 each annually as partial subsidy for using private primary care services. You do not need to pre-register, collect or carry the voucher."

The following are the views some people on the voucher scheme. Do you agree with the view?

		Strongly agree	Agree	Disagree	Strongly disagree	Don't know
11.	**11a. Do you agree "Voucher scheme is useful"?**<br><u>*(for those disagree i.e. not useful)*</u><br>**11b.** Why do you think the voucher scheme is not useful? Any more? *(Please check all that*	1	2	3 (Go to 11b)	4 (Go to 11b)	5

	apply)<br>1- Too little amount<br>2- Could not find an enrolled healthcare professionals nearby<br>3- The healthcare professionals whom I usually saw has not enrolled in the voucher scheme<br>4- Complicated procedure in using the voucher<br>5- I prefer using public healthcare services e.g. Government, HA services<br>6- Others, please specify:____________________					
12.	**12a. Do you agree "Voucher is convenient to use"?**<br>*(for those disagree i.e. inconvenient)*<br>**12b.** How to make it convenient to use? Any more? *(Please check all that apply)*<br>1- Use paper/ physical vouchers<br>2- More publicity on how to use it<br>3- Others, please specify: ____________________	1	2	3	4<br>(3–4: Go 12b)	5
13.	**Do you agree "Voucher scheme encourages you to use the private primary care services more than before"?**	1	2	3	4	5
14.	**14a. Do you agree "Voucher scheme does not change your behaviour on where to go to see the healthcare professionals?"**<br>*(for those agree i.e. does not change)*<br>**14b.** Why does it not change your behaviour? Any more? *(Please check all that apply)*<br>1- Too little amount<br>2- Could not find an enrolled healthcare professional nearby<br>3- The healthcare professionals whom I usually saw has not enrolled in the voucher scheme<br>4- Complicated procedure in using the voucher<br>5- Others, please specify: ____________________	1	2<br>(1–2: Go 14b)	3	4	5
15.	**15a. Do you agree "The amount of voucher of $250 per year is enough"?**<br>*(for those disagree i.e. not enough)*<br>**15b. For those elderly seeing private GPs/ specialists or visiting elderly health centres or those community elderly:** How much	1	2	3	4<br>(3–4: Go 15b or 15c)	5

	value of voucher per year do you think is appropriate? **(open-ended format)**<br>HK$______________________<br><br>**OR**<br>**15c. For those elderly recruited in GOPCs:**<br>How much value of voucher per year would make you go to see private doctors?<br>**(open-ended & bidding format)**<br>Increase the amount by HK$50 each time, say, HK$300, HK$350, HK400, … until the elderly says it will make them to see private doctors<br>HK$______________________					
16.	**16a. Do you agree "The coverage of healthcare services under the voucher scheme is sufficient"?**<br>*(for those disagree i.e. coverage not enough)*<br>**16b.** Whom do you want to add in the list of participating healthcare professionals e.g. optometrist, podiatrist, etc. or to add in what kind of additional services?<br>Please specify: ______________________	1	2	3	4	5
				Go 16b (3, 4)		
17.	**Do you agree "The voucher scheme has led to an increase in consultation fees"?**	1	2	3	4	5
	**Practice**					
	● ***Ever use***					
18.	**18a.** Have you <u>ever</u> used the voucher?	1- Yes (Go to Q19)<br>2- No (Skip to Q25 after asking Q18b)<br>3- Do not remember (Skip to Q25)				
	(For those not ever used the voucher)<br>**18b.** Why have you not ever used voucher? Any more?<br>*(Please check all that apply)*	1- I am not aware of the voucher scheme<br>2- Too little amount<br>3- Could not find an enrolled healthcare professional nearby<br>4- The healthcare professional whom I usually saw has not enrolled in the voucher scheme<br>5- Complicated procedure in using the voucher<br>6- I don't have to consult doctors/ other healthcare professionals<br>7- Others, please specify: ____________________				

19.	What triggers you to use the voucher? Any more? Any more?<br>*(Please check all that apply)*	1- Making good use of the subsidy<br>2- Shorter waiting time in seeing private healthcare professionals<br>3- Better quality in private sectors<br>4- Others, please specify: ________________
20.	**20a.** Usually, how many vouchers would you use per visit?<br><br>**20b.** Why do you use more than 1 voucher per visit? *(Please check all that apply)*	1- 1 piece of voucher → Go to Q21<br>2- 2 pieces<br>3- 3 pieces<br>4- 4 pieces<br>5- 5 vouchers<br>6- More than 5 vouchers, please specify: ____________<br>7- It depends<br><br>1- Use it now since I don't know when I will use it again<br>2- Healthcare professionals/clinic staff suggest using more than 1 voucher<br>3- I do not need to pay any cash<br>4- I only need to pay a small sum<br>5- Others, please specify: ________________
21.	Did the healthcare professional/clinic staff ever advise you of the number of vouchers that you should use during consultation?	1- Yes<br>2- No<br>3- Do not remember
22.	**22a.** Have you used all the vouchers $250 for the year 2009?<br><br>*<u>(for those who haven't used all 5 vouchers)</u>*<br>**22b.** Why do you not use all the vouchers for the year 2009? Any more?<br>*(Please check all that apply)*	1- Yes (Skip to Q23)<br>2- No<br><br>1- Hold it for future use<br>2- I do not need to use it<br>3- I prefer using public healthcare services e.g. HA services<br>4- I could not find an enrolled healthcare professionals nearby<br>5- Others, please specify: ________________
23.	What kind of <u>healthcare professionals</u> you have ever used for the voucher? Any more? (Read out one by one)<br>*(Please check all that apply)*	1- Registered medical practitioners<br>2- Registered Chinese Medicine practitioners<br>3- Registered dentists<br>4- Registered chiropractors<br>5- Registered nurses/ enrolled nurses<br>6- Registered physiotherapists<br>7- Registered occupational therapists

		8- Registered radiographers<br>9- Registered medical laboratory technologists
24.	What kind of <u>medical services</u> you have ever used for the voucher? Any more?<br>*(Please check all that apply)*	1- Acute curative services such as seeking medical advice/ treatment for acute diseases/ conditions<br>2- Regular follow up for chronic diseases/ conditions<br>3- Receive immunization<br>4- Undergo health checks/ screening<br>5- Rehabilitative services<br>6- Others, please specify: ____________________
	● ***Healthcare services utilization & voucher use***	
25.	Did you usually go to public or private doctors for consultation **before** the launch of voucher scheme?	1- Public doctor<br>2- Private doctor<br>3- Both<br>4- Don't know
26.	In the <u>past 1 month</u>, how many times have you consulted doctors or other healthcare professionals? Including doctors in emergency units and government clinics, private practitioners, Chinese medicine practitioners, dentists, physiotherapists, etc.	1- No (Go to Q30)<br>2- Yes (Total frequency: ___________)
	**<u>Probe</u>:** How many times have you consulted	______ times
	(a) Practitioners of western medicine in Emergency Units	______ times
	(b) Practitioners of western medicine in Government clinics (including general and specialist)	______ times
	(c) Private practitioners of western medicine (including general and specialist)	______ times
	(d) Chinese medicine practitioners (including acupuncturists, herbalists and bonesetters)	
	(e) Other healthcare professionals e.g. dentists, physiotherapists, etc.<br>Please specify: ________________	______ times

27. *(For those who have consulted Q26c <u>private practitioners of western medicine</u>)*	
(a) **Have you used the voucher for <u>the LAST consultation of private western medicine practitioner</u>?**	1- Yes<br>2- No ⟶ Go to Q27f
(b) How many vouchers have you used?	No. of vouchers: _____ Total value: HK$ _________
(c) What is the reason/ purpose for consulting the doctor?	1- Seek medical advice/ treatment for acute diseases/ conditions (e.g. colds, acute injuries) or discomfort/ symptoms<br>2- Regular follow-up for chronic diseases/ conditions<br>3- Receive immunization<br>4- Undergo health checks/ screening<br>5- Others, please specify:_____________________
(d) How much did you pay to the doctor for the total charges for consultation, medicine, etc. excluding the voucher amount?	HK$_______________
(e) Did the amount (27b + 27d) more/ less/ the same as the consultation fee you usually paid in the past?	1- More<br>2- Less<br>3- The same<br>4- Don't know
(f) Why did you not use the voucher?	Reasons: _____________________________

28. *(For those who have consulted Q26d <u>Chinese medicine practitioners</u>)*	
(a) Were you consulting **<u>private</u>** <u>Chinese medicine practitioners</u> at last consultation?	1- Yes<br>2- No → Go to Q29
**(b) Have you used the voucher for the LAST consultation of private Chinese medicine practitioner?**	1- Yes<br>2- No → Go to Q28g
(c) How many vouchers have you used?	No. of vouchers: _____ Total value: HK$ _________
(d) What is the reason/ purpose for consulting the doctor?	1- Seek medical advice/ treatment for acute diseases/ conditions (e.g. colds, acute injuries) or discomfort/ symptoms<br>2- Regular follow-up for chronic diseases/ conditions<br>3- Undergo health checks/ screening<br>4- Others, please specify:_____________________
(e) How much did you pay to the Chinese medicine doctor for the total charges for consultation, medicine, etc. excluding the voucher amount?	HK$_______________
(f) Did this amount (28c+28e) more/ less/ the same as the consultation fee you usually paid in the past?	1- More<br>2- Less<br>3- The same<br>4- Don't know
(g) Why did you not use the voucher?	Reasons: _____________________________

29.	*(For those who have consulted Q26e <u>other healthcare professionals</u>)*	
	(a) What kind of healthcare professionals e.g. dentists, physiotherapists have you consulted last time?	Healthcare professionals: ____________________
	(b) Were you consulting the healthcare professionals in the **<u>private</u>** <u>sector</u> at last consultation?	1- Yes<br>2- No ⟶ Go to Q30
	**(c) Have you used the voucher for the LAST consultation with the dentist?**	1- Yes<br>2- No ⟶ Go to Q29h
	(d) How many vouchers have you used?	No. of vouchers: _____ Total value: HK$ _________
	(e) What is the reason/ purpose for consulting the dentist?	1- Seek medical advice/ treatment for acute conditions (e.g. teeth pain)<br>2- Regular check-up<br>3- Rehabilitation<br>4- Others, please specify:____________________
	(f) How much did you pay to the dentist for the total charges for consultation, medicine, etc. excluding the voucher amount?	HK$______________
	(g) Did this amount (29d+29f) more/ less/ the same as the consultation fee you usually paid in the past?	1- More<br>2- Less<br>3- The same<br>4- Don't know
	(h) Why did you not use the voucher?	Reasons: ___________________________
30.	Have you been admitted into hospitals in Hong Kong <u>in the past 1 year</u>?	1- Yes (Probe: No. of hospitalization: __________)<br>2- No
	**Respondent's profile**	
	● ***Demography & socioeconomic status***	
31.	Gender	1- Female<br>2- Male
32.	What is your current marital status?	1- Single<br>2- Married<br>3- Divorced / Separated

		4- Widowed
33.	Including yourself and live-in domestic helper, how many persons are living with you? I refer to those who stay overnight here for at least four days a week on average.	Number of persons: __________________<br><br>**If living alone, please skip to Q35.**
34.	What are your relationships with these household members? Any more?<br>*(Please check all that apply and* ***record the number for each type of relatives****)*	Number:<br>1- Husband/ wife ______<br>2- Children ______<br>3- Daughters-in-law/ Sons-in-law ______<br>4- Grandchildren ______<br>5- Brothers/ Sisters ______<br>6- Other relatives ______<br>7- Others: Please specify ______
35.	Which district do you live?	**Hong Kong Island**<br>1- Central & Western<br>2- Eastern<br>3- Southern<br>4- Wan Chai<br>**Kowloon**<br>5- Kowloon City<br>6- Kwun Tong<br>7- Sham Shui Po<br>8- Wong Tai Sin<br>9- Yau Tsim Mong<br>**New Territories**<br>10- Island<br>11- Kwai Tsing<br>12- North<br>13- Sai Kung<br>14- Sha Tin<br>15- Tai Po<br>16- Tsuen Wan<br>17- Tuen Mun<br>18- Yuen Long
36.	What is your highest educational attainment?	1- No formal education or Kindergarten<br>2- Primary<br>3- Lower secondary (F.1 – F.3)<br>4- Upper secondary (F.4 – F.5)<br>5- Matriculation<br>6- Technical / Vocational training<br>7- Tertiary or above

37.	Are you working?	1- Yes<br>2- No
38.	Which of the following sources of income do you have/ or do you receive any kind of financial support? Any more? (Read out one by one)<br>*(Please check all that apply)*	1- Job income/ Salary<br>2- Pension<br>3- Financial support from children/ other relatives<br>4- CSSA<br>5- Disability allowance<br>6- Old Age Allowance<br>7- Other sources of income (e.g. interests, bonus, rental income, etc.)<br>8- No income
39.	Including all sources of income and MPF contribution, how much is your monthly household income approximately?	Amount: HK$_______________<br>1- HK$1-$4,999<br>2- HK$5,000 to $9,999<br>3- HK$10,000 to $14,999<br>4- HK$15,000 to $19,999<br>5- ≥HK$20,000<br>6- No income<br>7- Not willing to answer<br>8- Don't know
40.	**40a.** Do you have any medical benefits/ health insurance?	1- Yes (Go to Q40b)<br>2- No (Skip to Q41)
	(For those who have medical benefits/ health insurance)<br>**40b.** What does the medical benefits/ health insurance cover? Any more?<br>*(Please check all that apply)*	1- Hospitalization<br>2- General out-patient<br>3- Specialist out-patient<br>4- Dental consultation<br>5- Chinese herbalist/ acupuncturist/ bonesetter<br>6- Annual medical check-up<br>7- Provides a fixed amount of reimbursement (not limited to specific item)<br>8- Others, please specify _______________________<br>9- Don't know/ can't remember
	● ***Disease diagnose***	
41.	Have you ever been told by a western medicine practitioner that you had the following chronic health conditions?<br>*(Read out the answer one by one and then check all that apply)*	1- Cancer<br>2- Diabetes mellitus<br>3- High blood pressure<br>4- Heart diseases<br>5- Stroke

		6- Asthma<br>7- High cholesterol<br><br>**8- None of the above**
42.	Do you have other chronic health conditions as told by a Western medicine practitioner, but not mentioned in the list above?<br>*(<u>Do not read out</u> the answer one by one, just check those answers mentioned by respondents)*	**Diseases of the blood**<br>1- Anaemia<br>2- Congenital blood disease (e.g. thalassemia, hemophilia)<br>3- Others, please specify: ________________<br>4- Immune disease (e.g. SLE, Rheumatoid arthritis)<br>**Endocrine & metabolic diseases**<br>5- Thyroid disease<br>6- Others, please specify:____________________<br>**Mental disorder**<br>7- Depression<br>8- Anxiety disorder<br>9- Schizophrenia<br>10- Dementia<br>11- Others, please specify: ____________________<br>**Diseases of the nervous system**<br>12- Epilepsy<br>13- Parkinson's disease<br>14- Others, please specify:_____________________<br>**Disease of the eye**<br>15- Disease of the eye (e.g. glaucoma, cataract, bad vision, blindness)<br>**Diseases of the ear/ nose/ throat (ENT)**<br>16- Diseases of the ear/ nose/ throat (e.g. sinusitis allergic rhinitis, hearing loss, tinnitus)<br>**Diseases of the heart or circulatory system)**<br>17- Diseases of the heart or circulatory system<br>18- Others, please specify:_____________________<br>**Respiratory diseases**<br>19- Emphysema, chronic bronchitis, bronchiectasis<br>20- Tuberculosis<br>21- Others, please specify:_____________________<br>**Stomach & intestinal disease**<br>22- Stomach & intestinal disease (e.g. gastric ulcer)<br>**Liver disease**<br>23- Liver disease (e.g. hepatitis B or C, cirrhosis)<br>**Skin disease**<br>24- Skin disease (e.g. eczema, psoriasis)

		**Musculoskeletal disease**<br>25- Arthritis, rheumatism<br>26- Low back pain<br>27- Gout, high uric acid<br>28- Osteoporosis<br>29- Others, please specify:______________________<br>**Kidney disease**<br>30- Kidney disease (e.g. kidney failure, nephritis, nephrosis, requiring dialysis)<br>**Complications of previous injury**<br>31- Complications of previous injury (e.g. loss of function of limb(s))<br>32- Others, please specify:______________________<br><br>**33- None of the above**
43.	How many chronic pharmaceutical treatment that you are receiving?	1- Not applicable (No need to take pharmaceutical treatment)<br>2- 1-4 type<br>3- 5 type or above<br>4- Don't know
	● ***Self-rated health***	
44.	Comparing with other persons of your same age, what do you think about your health condition? (read out)	1- Much better<br>2- Better<br>3- Similar<br>4- Worse<br>5- Much worse
45.	Comparing with last year, what do you think about your general health condition now? (read out)	1- Much better than last year<br>2- Better than last year<br>3- Similar to last year<br>4- Worse than last year<br>5- Much worse than last year
	● ***Life style***	
46.	Do you smoke?	1- Yes<br>2- No (Skip to Q48)
47.	*(for smokers)*<br>(a) How many days do you smoke in a week?<br><br>(b) How many cigarettes do you smoke in a	 1- Every day<br>2- 3 to 6 days a week<br>3- 1 to 2 days a week<br>4- Less than 1 day in a week<br><br>Number of cigarettes per day: ______________

	day on average?	
48.	*(for non-smokers)*<br>Did you smoke in the past?	1- Used to smoke everyday, but have already quitted smoking<br>2- Used to smoke occasionally, but have already quitted smoking<br>3- Have never smoked
49.	Do you have a habit of drinking alcohol?	1- Yes<br>2- No (Skip to Q51)
50.	*(for drinkers)*<br>(a) On average, approximately how many days per week do you drink?	1- Every day<br>2- 4 – 6 days per week<br>3- 1 – 3 days per week<br>4- 1 – 3 days per month<br>5- Not even once a month
	(b) What type of alcoholic beverage you mainly drink?	1- Beer<br>2- Black beer<br>3- Wine (red wine, white wine)<br>4- Liquor (brandy, whisky, etc.)<br>5- Cocktail<br>6- Chinese Rice Wine<br>7- Japanese Sake<br>8- Others: please specific ____________________
51.	*(for non-drinkers)*<br>Is that you never drink, or used to drinking but now quitted, or do you only drink on special occasions?	1- Never drink<br>2- Used to drink, but now quitted<br>3- Only drink on special occasions
52.	How many fruits do you take in a day on average? (One fruit equal to an average size orange or pear)	1- Do not eat fruit<br>2- Less than 1<br>3- 1<br>4- 2<br>5- 3 or above
53.	How many bowls of vegetables do you take everyday? (Based on one average rice bowl of 300 ml)	1- Seldom<br>2- Less than half bowl<br>3- Half to one bowl<br>4- More than one bowl
54.	Do you do exercise regularly?	1- Yes<br>2- No (Skip to Q56)
55.	*(for those who do exercise regularly)*<br>(a) How many days do you do exercise in a	1- Everyday

	week?	2- 3 to 6 days a week<br>3- 1 to 2 days a week<br>4- Less than 1 day in a week
	(b) How long do you do exercise every time?	____ hr ____min
	(c) What type of exercise do you usually do? Any more?<br>*(Please check all that apply)*	1- Tai Chi<br>2- Qi-gong<br>3- Morning-walk<br>4- Jogging<br>5- Swimming<br>6- Luk Tung Kuen<br>7- Others, please specify ________________________
56.	*(for those who do not exercise regularly)*<br>What are the reasons for not doing exercise? Any more? *(Please check all that apply)*	1- No time/ too busy<br>2- Health condition can't afford/ illness/ tired<br>3- No one accompanied me<br>4- No facilities/ no space<br>5- Don't know how to exercise<br>6- Don't want to spend money<br>7- Others: please specify ___________________

End of the Questionnaire

This is a longitudinal study. We wish to seek your opinion again in one year later. Might you leave your telephone number to us such that we could contact you later?

Telephone number: ___________________ Name: __________________________

Thank for your much for your participation in the Survey!
